# Age-Specific Modulation of Prefrontal Cortex LTP by Glucocorticoid Receptors Following Brief Exposure to HFD

**DOI:** 10.3389/fnsyn.2021.722827

**Published:** 2021-10-04

**Authors:** Kuldeep Shrivastava, Tali Rosenberg, Noam Meiri, Mouna Maroun

**Affiliations:** ^1^Sagol Department of Neurobiology, Faculty of Natural Sciences, University of Haifa, Haifa, Israel; ^2^Agricultural Research Organization, The Volcani Center, Institute of Animal Science, Rishon LeZion, Israel

**Keywords:** prefrontal cortex, LTP, juvenile, plasticity, glucocorticoids, adult, basolateral amygdala

## Abstract

The corticolimbic circuits in general and the medial prefrontal cortex in particular, undergo maturation during juvenility. It is thus expected that environmental challenges in forms of obesogenic diet can exert different effects in juvenile animals compared to adults. Further, the relationship between glucocorticoids and obesity has also been demonstrated in several studies. As a result, glucocorticoid receptor (GR) antagonists are currently being tested as potential anti-obesity agents. In the present study, we examined the effects of short-term exposure to high-fat diet (HFD) on prefrontal long-term potentiation (LTP) in both juvenile and adult rats, and the role of glucocorticoid receptors (GRs) in modulating these effects. We found HFD impaired prefrontal LTP in both juveniles and adults, but the effects of GR modulation were age- and diet-dependent. Specifically, GR antagonist RU-486 reversed the impairment of LTP in juvenile animals following HFD, and had no effect on control-diet animals. In adult animals, RU-486 has no effect on HFD-impaired LTP, but abolished LTP in control-diet animals. Furthermore, impairments in the prefrontal LTP following HFD are involved with an increase in the mPFC GR levels only in the juveniles. Further, we found that *in vivo* application of GR agonists into adult mPFC rescued HFD-induced impairment in LTP, suggesting that these receptors might represent strategic therapeutic targets to potentially combat obesity and metabolic related disorder.

## Introduction

Overconsumption of a Western diet rich in saturated fat and sugar is recognized as one of the primary risk factors for the development of obesity and associated metabolic disorders (Vickers et al., 2005; Francis and Stevenson, 2013). There is also growing evidence of direct adverse effects of exposure to obesogenic diets on emotional regulation, anxiety-like behaviors, and cognitive processes (Ortolani et al., 2011; Francis and Stevenson, 2013; Boitard et al., 2015; Reichelt et al., 2015b; Sivanathan et al., 2015; Baker and Reichelt, 2016; Kalyan-Masih et al., 2016; Vega-Torres et al., 2018). Research has focused primarily on the detrimental impact of diet on hippocampus-based cognitive functions (Boitard et al., 2012; Reichelt et al., 2015b; Khazen et al., 2019)]. However, emerging studies in rodents and humans have shown the impact of a high-fat diet (HFD) to extend also to other brain regions, such as the medial prefrontal cortex (mPFC) (Edwards et al., 2011; Kanoski and Davidson, 2011; Bocarsly et al., 2015; Yaseen et al., 2018).

The corticolimbic pathway which connects the mPFC, amygdala and the hypothalamic-pituitary-adrenal (HPA) axis undergoes dramatic structural reorganization during juvenility and/or adolescence, which is a critical period for brain maturation (Boitard et al., 2015; Arruda-Carvalho et al., 2017; Silvers et al., 2017). Recent findings suggest that this corticolimbic pathway is negatively impacted by an obesogenic diet during adolescence (Vega-Torres et al., 2018). Recently, it has been shown in rats that 6 weeks of HFD or sugar consumption, especially if starting at adolescence (postnatal day; PND 32–45), has strong adverse effects on prefrontal-dependent cognitive functions (Baker et al., 2014; Reichelt et al., 2015b; Morin et al., 2017; Labouesse et al., 2018). Recently, we reported that short- and long-term exposure to HFD during juvenility (PND 27–29) impaired emotional memory, cognitive functions, long-term potentiation (LTP) in the hippocampus and the amygdala along with dysregulation of the HPA axis (Boitard et al., 2015; Khazen et al., 2019).

Previous studies have shown that HFD during adolescence is associated with several changes at the cellular and synaptic scales within the mPFC but had no effect in adulthood (Labouesse et al., 2018). However, only few studies focused on the acute effects of exposure to HFD on the juvenile brain (Yaseen et al., 2018; Khazen et al., 2019). Glucocorticoids act as a neuromodulator and have a critical role in regulating energy metabolism (Kalafatakis et al., 2016) and neuronal plasticity (Garabedian et al., 2017). Moreover, under psychological and physiological stressors, the HPA axis triggers the neuro-endocrine stress response resulting in a massive adrenal release of cortisol and the regulation of carbohydrate, lipid and protein metabolism (Christiansen et al., 2007; Di Dalmazi et al., 2012). High concentrations of glucocorticoids then induce a negative feedback loop on the HPA axis to stop the stress response and maintain the homeostasis of the system (Harrell et al., 2016).

To the best of our knowledge, the effects of HFD on mPFC plasticity and the modulation of glucocorticoid receptors (GRs) have not been studied in juvenile animals. In the present study, we sought to compare the effects of short-term HFD exposure on LTP in the basolateral amygdala (BLA)-mPFC pathway in juveniles and adults, and to address the role of GRs in the modulation of acute HFD-induced LTP. We found that brief exposure to HFD impaired mPFC-dependent LTP in juveniles and adults; however, these effects were differently modulated by GRs in juveniles and adults. Distinctively, systemic or local inhibition of GRs rescued prefrontal LTP in HFD-fed juveniles, but did not affect HFD-fed adults.

## Materials and Methods

### Animals and Diets

The experiments were performed using juvenile (21–28 days old) and adult (60–67 days old) male rats from the local animal colony at Haifa University. All experimental procedures and protocols were approved by the Ethics Committee of Haifa University for Experimentation with Animals and were performed in strict accordance with University of Haifa animal ethics regulations. The juvenile animals were separated from the dam on PND 21. A maximum of two animals from the same litter were used per experiment. Animals were housed in Plexiglas cages (4–5 rats per cage) and were maintained on a free feeding regimen and a 12 h light/dark cycle. All experiments were performed in the light phase, from 9 am to 1 pm. From PND 21 to PND 28 for juveniles, and from PND 60 to PND 67 for adults. In rats, adolescence begins around PND 28 and ends at approximately PND 50–55 when rats are reproductively mature, indicating the transition into adulthood, as this bypasses most known behavioral and physiological changes associated with adolescence (Spear, 2000). We consistently use this age in our and other’s research and thus, this enables us to compare with other studies (Sasaki et al., 2013; Boitard et al., 2015; Khazen et al., 2019). We specifically avoid using weight range to define the onset of adulthood. Several studies that use *in vitro* recording, slices are taken from the brains of 200–250 gr rat which may still be undergoing adolescence. Animals were exposed either to the regular standard chow diet (CD; ENVIGO, Israel) or to HFD (D12492, Research Diets, New Brunswick, NJ, United States). CD offers 3 kcal/g and consists of 4% fat and 60% carbohydrate (35% kcal), whereas HFD offers 5.2 kcal/g and consists of 35% fat, mostly saturated fat from lard (60% kcal), and 26% carbohydrate (20% kcal). Electrophysiological experiments were conducted on the last day of the animals’ respective diets (PND 28 for juveniles, PND 67 for adults).”

### Electrophysiology

**FIGURE 1:**
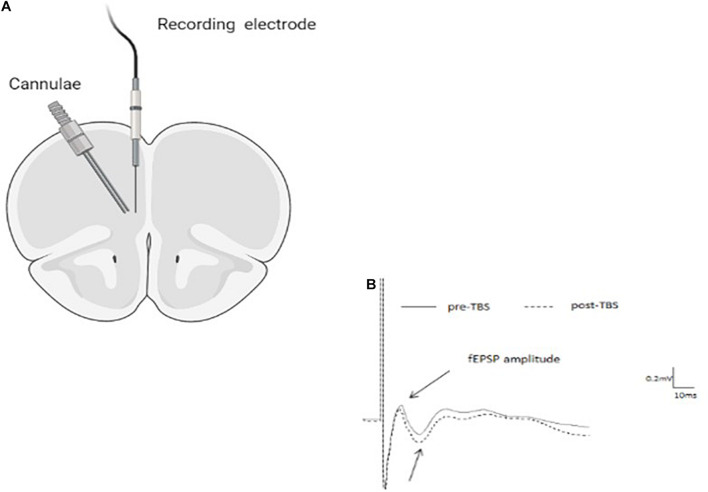
**(A)** Schematic drawing indicating cannula and recording electrode positions within the infra-limbic medial prefrontal cortex (IL-mPFC). **(B)** Representative average waveforms before and after TBS.

Juvenile and adult male rats were anesthetized with a mixture of urethane and chloral hydrate [40% urethane, 5% chloral hydrate in saline; 0.5 ml/100 g intraperitoneal (i.p.) and with supplementary injections (0.1–0.2 ml) as necessary to ensure full anesthesia] and placed in a stereotaxic frame (Stoelting, United States). Body temperature was maintained at 37 ± 0.5°C. Small holes were drilled into the skull to allow the insertion of electrodes into the brain. A single recording microelectrode (glass tip diameter 2–5 μm; filled with 2M NaCl; resistance 1–4 Ω) was slowly lowered into the infralimbic (IL)- mPFC—for juveniles: anteroposterior 2.7–2.9 mm anterior to bregma, 0.4–0.6 mm lateral, 3.4–3.8 mm below the pial surface; for adults: anteroposterior 3.0–3.3 mm anterior to bregma, 0.7–1.0 mm lateral, 3.8–4.8 mm below the pial surface (Figure 1).

A bipolar 125 μm stimulating electrode was inserted into the BLA: for adults: anteroposterior −3 mm relative to bregma, lateral 5 mm, ventral −7.6 mm; for juveniles: anteroposterior −2.6 mm relative to bregma, lateral 5.0 mm, ventral −7.4 mm (Schayek and Maroun, 2015; Yaseen et al., 2018). The evoked responses were digitized (10 kHz) and analyzed using a Cambridge Electronic Design 1,401 Plus Data Acquisition system (Cambridge, United Kingdom) and its Spike2 software. Offline measurements of field postsynaptic potential (fEPSP) amplitude used averages of five successive responses to Test stimuli (monopolar pulses; 100 μs duration) were delivered at 0.1 Hz. After positioning the electrodes, rats were left for 30 min before commencing the experiment.

### LTP Induction

Long-term potentiation was induced using Theta Burst Stimulation (TBS) protocol, which was applied in all groups within 1.5 h of anesthesia. TBS was delivered at BLA in three sets of 10 trains, each train consisting of 10 pulses at 200 Hz for juvenile animals and at 100 Hz for adult animals, with inter-train interval 200 ms and inter-set interval 1 min. We aimed to use similar protocols of TBS for the induction of LTP in both juvenile and adult animals; however, consistently with previous reports, the 100 Hz TBS protocol failed to induce potentiation in juvenile controls (Schayek and Maroun, 2015; Khazen et al., 2018) while it induced LTP in adult controls. As a result, different TBS protocols were used in the two age groups to induce similar and comparable levels of potentiation in both groups (Khazen et al., 2018).

Theta Burst Stimulation was delivered at the same intensity and pulse duration as the test stimuli during establishment of the baseline responses. Evoked fEPSPs at the baseline intensity were recorded from the IL-mPFC for up to 60 min following the application of TBS. The results are presented as the mean of the preceding 5 min period to give 12 time points. Changes in fEPSPs amplitude were measured for each rat as a percentage of change from its baseline. LTP was defined as an increase in the amplitude of the fEPSPs.

### Drugs

The GR antagonist RU-486 (RU; Sigma) was first dissolved in 100% ethanol and subsequently diluted in saline to reach the appropriate concentration of 10 mg/kg, based on previous results (Segev et al., 2012). The final ethanol concentration was 1%. Controls were given the vehicle (1% ethanol) only. The selective GR agonist RU-28362 (11β, 17β-dihydroxy-6, 21-dimethyl-17α-pregna-4, 6-trien-20-yn-3-one), first dissolved in 100% ethanol and subsequently diluted with saline to reach its appropriate concentration, was infused at a 10ng dose (adapted from Roozendaal et al., 2004). For electrophysiology, RU-486/RU-28362/vehicle was injected 30 min before TBS (Boitard et al., 2015; Khazen et al., 2019).

### Microinjection

Rats were anesthetized and placed in a stoelting stereotaxic frame, and microinjected with 10 ng RU-486 (in 0.5 μl) into the IL-mPFC (Figure 1). For microinjection, a stainless steel guide cannula (23 gauge, thin wall) was lowered into the IL-mPFC and a 28-gauge injection cannula, extending 1.0 mm from the tip of the guide cannula, was inserted through it. The injection cannula was connected *via* PE20 tubing to a Hamilton micro syringe driven by a micro infusion pump (PHD 100, Harvard Apparatus). Microinjection of the 0.5 μl volume was delivered over 2 min. The injection cannula was left in position for an additional 2 min before withdrawal in order to minimize dragging of the injected liquid along the injection tract. Intra-mPFC injections were performed 30 min before the application of TBS.

### Histology

Upon completion of recording animals were sacrificed and brains were taken for histology. In cannulated animals, Indian ink (0.5 μl) was microinfused into the IL and brains were stored at −80°C. Coronal sections of 30 μm were cut using a cryostat. Following Nissl staining, placement of cannulae and electrodes were observed under a light microscope. Figure 1 shows a schematic representation of the placement of cannulae in the IL (coronal view at position +3.10 and +2.70 mm anterior to bregma). Arrow indicates the locations. Animals that did not have the tips of their cannulae and/or electrodes in both IL and the BLA were omitted from analysis (overall 4).

### Brain Sections

Frozen coronal brain sections of the PFC were cut using a cryostat (2.7 to 2.9 mm from bregma in juveniles and 3.0–3.3 mm in adults (Paxinos and Watson, 1998). The PFC was extracted using a truncated needle (32-gauge diameter). The PFC punches were either immersed in RNA later (Ambion, Austin, TX, United States) or frozen in dry ice.

### RNA Isolation and Real-Time Polymerase Chain Reaction

RNA was isolated and reverse transcribed, as described previously (Marcia and Pyle, 2014). Real-time polymerase chain reaction (qPCR) was performed, in duplicates, with 200 ng complementary DNA in a step-one plus sequence analysis system (Applied Biosystems, Foster City, CA, United States) with Perfecta qPCR Super Mix (Quanta bio, Beverly, MA, United States). *Hprt1* was used as a reference gene for reverse transcription PCR (RT-PCR), The primers were (5′→3′): *GR* (NM_012576.2) forward: GGAAGGTCTGAAGAGCCAAG, reverse: TTCCCTTTTGACGATGGC.*Hprt1* (NM_012583.2) forward: GCGAAAGTGGAAAAGCCAAG, reverse: GCCACATCAACAGGACTCTTGTAG. Dissociation curves were analyzed after each RT-PCR cycle to confirm the presence of only one product and the absence of primer-dimer formation. The threshold cycle (Ct) for each tested gene (*X*) was used to quantify the relative abundance of that gene using the livak delta delta CT formula: 2 ^–(Ct gene^ *^*X*^*^– *Ct standard)*^.

### Statistical Analysis

Data were expressed as the mean ± standard error of the mean (SEM), and statistical analyses were conducted using SPSS (version 2.1) or Prism 7 software with the threshold for significance set at *p* ≤ 0.05. Body weight data were analyzed using unpaired Student’s *t* tests. LTP results were analyzed using analysis of variance (ANOVA) with Diet (CD vs HFD), Age (juvenile vs adult), or Drug treatment (RU-486/RU-28362 vs vehicle) as between-subject factors and with repeated measurement on the time factor when appropriate. Differences in mRNA levels (RT-qPCR) between the HFD and CD animals were analyzed using univariate ANOVA analysis. Significant interactions were further investigated using Tukey’s *post hoc* tests

## Results

### Experiment 1: 7-day HFD Exposure Does Not Affect Body Weight in Juveniles or Adults

**FIGURE 2:**
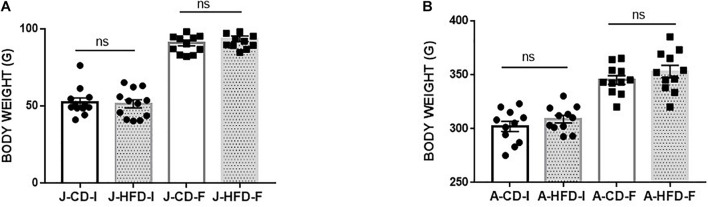
Body weight gain during 7-day high-fat diet (HFD) exposure. Juvenile [J; post-natal day (PND) 21–28] and adult (A; PND 60–67) animals body weight was measured immediately prior to and immediately after completing a 7-day HFD or standard chow diet (CD) regimen. The change in body weight showed no significant effect of Diet (n.s) or of Age × Diet interaction (n.s). The group were following: **(A)** HFD fed animals [Juvenile-Initial (J –HFD-I), Juvenile-Final (J-HFD -F), *N* = 12 and Adult-Initial (A-HFD-I), Adult-Final (A-HFD-F), *N* = 11]. **(B)** CD-fed animals [Juvenile-Initial (J –CD- I), Juvenile-Final (J-CD-F), *N* = 12 and Adult-Initial (A–CD-I), Adult-Final (A-CD-F), *N* = 11]. Error bars show standard error of the mean (SEM).

We examined the impact of 7-day HFD consumption on body weight change during this time (Figure 2). In both juveniles and adults, we did not observe any significant differences in body weight change between HFD-fed and CD-fed animals [*F*(1, 23) = 0.755, n.s.], nor was there an interaction of Age × Diet: [*F*(1, 23) = 1.422], suggesting that the HFD diet regimen does not induce obesity on this time scale.

### Experiment 2: 7-Day HFD Exposure Impairs Prefrontal LTP in Juveniles and Adults

**FIGURE 3:**
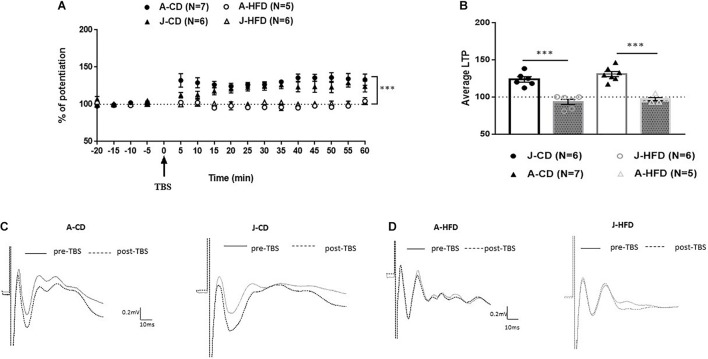
Effect of 7-day HFD exposure on prefrontal long-term potentiation (LTP) in juvenile and adult animals. Electrophysiological recordings were performed on juvenile (PND 28) and adult (PND 67) animals immediately after completing a 7-day HFD or CD regimen. **(A)** Normalized field excitatory post-synaptic potentials (fEPSPs) recorded *in vivo* before and after TBS of the BLA-mPFC pathway. Each data point shows the average fEPSPs of the preceding 5 min. **(B)** Average LTP shown in panel A over the 60 min after TBS. **(C,D)** Representative average waveforms before and after TBS Horizontal bar = 10 ms; vertical bar = 0.2 mV. Overall: HFD was shown to impair LTP in juveniles and adults: Two-way ANOVA with repeated measures on the 12 post-TBS time points showed a significant effect for Diet (^∗∗∗^*p* < 0.001) without a significant effect of Age × Diet interaction. Error bars show standard error of the mean (SEM).

We first assessed how 7-day exposure to HFD during juvenility and adulthood would affect mPFC-LTP (Figure 3). The groups were as following [J-CD (*n* = 6), J-HFD (*n* = 6); A-CD (*n* = 7), A-HFD (*n* = 5)].

The baseline amplitude and the stimulation intensity required to produce comparable baseline signals were not affected by Age, Diet, or Drug [*F*(1, 20) < 1]. Two-way ANOVA with repeated measures on the 12 time points following TBS {Diet (CD, HFD) × Age [adult (A), juvenile (J)]} revealed a significant effect for Diet [*F*(1, 20) = 56.022; *p* < 0.001], without effects of Age [*F*(1, 20) = 0.527, n.s.] or Age × Diet interaction [*F*(1, 20) = 3.240, n.s.]. These results indicate that 7-day HFD exposure reduces LTP in the mPFC in both juveniles and adults.

### Experiment 3: Systemic GRs Antagonist Rescues HFD-Induced LTP Impairment in Juveniles but Had no Effect in Adults

We next sought to examine whether systemic blockade of GRs can rescue LTP in HFD-fed animals. Systemic injections were performed prior to TBS. Eight groups were examined: juvenile and adult animals that were placed on CD or HFD and received either vehicle (VEH) (J-CD-VEH, *n* = 5; J-HFD-VEH, *n* = 5; A-HFD-VEH, *n* = 4; A-CD-VEH, *n* = 5) or RU-486 injections (J-CD-RU, *n* = 6; J-HFD-RU, *n* = 6; A-CD-RU, *n* = 5; A-HFD-RU, *n* = 6).

The baseline amplitude and the stimulation intensity required to produce comparable baseline signals were not affected by Age, Diet, or Drug [*F*(1, 34) < 1].

Analysis of variance with repeated measures {Age (A,J), Diet (CD, HFD), and Drug [vehicle (VEH), RU-486]} on the 12 post-TBS time points showed significant effects of Diet [*F*(1, 34) = 18.514, *p* < 0.001], Age [*F*(1, 34) = 14.356, *p* < 0.001], and Diet × Drug interaction [*F*(1, 34) = 9.048, *p* < 0.005], without an effect of Drug [*F*(1, 34) = 0.713, n.s]. To investigate the interaction at each age and to pinpoint its source, each age was analyzed separately.

**FIGURE 4:**
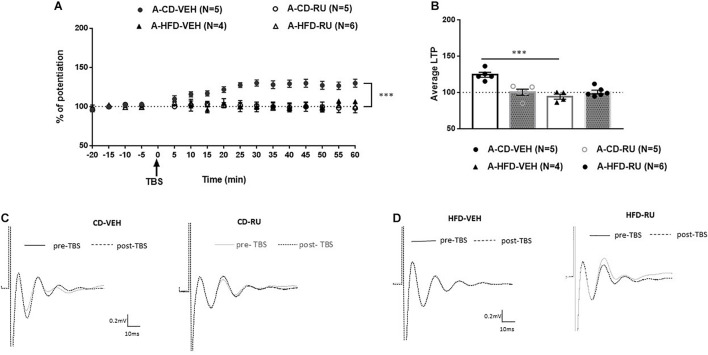
Effects of systemic glucocorticoid receptor (GR) blockade on prefrontal LTP in HFD- and CD-fed adults. **(A)** Normalized field excitatory post-synaptic potentials (fEPSPs) recorded *in vivo* before and after TBS of the BLA-mPFC pathway. Each data point shows the average fEPSPs of the preceding 5 min. **(B)** Average LTP shown in panel **(A)** over the 60 min after TBS. **(C,D)** Representative average waveforms before and after TBS Horizontal bar = 10 ms; vertical bar = 0.2 mV. For adult animals systemically injected with GR antagonist RU-486 (RU) or vehicle (VEH) 30 min prior to TBS. RU treatment had no effect on HFD-impaired LTP in HFD-fed animals, but impaired LTP in control CD-fed animals. Two-way ANOVA with repeated measures on the 12 post-TBS time points showed significant effects of Diet (^∗∗∗^*p* < 0.001), Drug (^∗∗∗^*p* < 0.005), and Diet × Drug interaction (^∗∗∗^*p* < 0.001). *Post hoc* analysis showed a significant difference in CD-fed animals between vehicle and RU-486 groups (^∗∗∗^*p* < 0.001), and no significant difference between HFD-treated groups. Error bars show standard error of the mean (SEM).

In adults, ANOVA with repeated measures indicated significant effects of Diet [*F*(1, 16) = 10.928, *p* < 0.005], Drug [*F*(1, 16) = 14.342, *p* < 0.001] and Diet × Drug interaction [*F*(1, 16) = 12.758, *p* < 0.005; Figure 4]. *Post hoc* analysis showed a significant difference between RU-486- and CD-VEH groups [*F*(1, 8) = 19.854, *p* = 0.002]. Specifically, whereas, the CD-VEH group showed intact potentiation, RU-486 treated group did not show potentiation [A-CD-VEH (124.3 ± 2.4%); A-CD-RU (100.2 ± 3.39%)]. In HFD fed animals, no differences were observed between vehicle and RU-486 [*F*(1, 8) = 0.046; n.s] and both groups did not show any potentiation [A-HFD-VEH (95.2 ± 4.4%); A-HFD-RU (99.6 ± 3.9%)].

**FIGURE 5:**
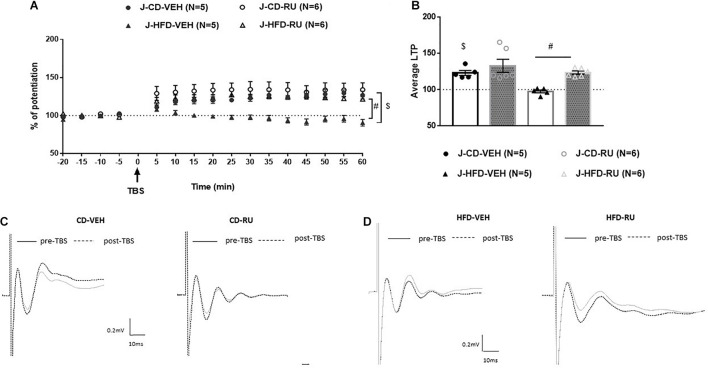
Effects of systemic GR blockade on prefrontal LTP in HFD- and CD-fed juveniles. **(A)** Normalized fEPSPs recorded *in vivo* before and after TBS of the BLA-mPFC pathway. Each data point shows the average fEPSPs of the preceding 5 min. **(B)** Average LTP shown in panel **(A)** over the 60 min after TBS. **(C,D)** Representative average waveforms before and after TBS Horizontal bar = 10 ms; vertical bar = 0.2 mV. For juvenile animals systemically injected with RU-486 (RU) or vehicle (VEH) 30 min prior to TBS. RU treatment rescued HFD-impaired LTP in HFD-fed animals, and had no effect on control CD-fed animals. Two-way ANOVA with repeated measures on the 12 post-TBS time points showed significant effects of Diet (^$^*p* < 0.001) and Drug (^#^*p* < 0.001), without a significant Diet × Drug interaction. Error bars show standard error of the mean (SEM).

In juveniles, ANOVA with repeated measures showed significant effects of Diet [*F*(1, 18) = 10.117, *p* < 0.005] and Drug [*F*(1, 18) = 10.799, *p* = 0.004] without a significant interaction [*F*(1, 18) = 2.197, n.s; Figure 5]. Both the J-CD-VEH and J-CD-RU groups show intact potentiation [J-CD-VEH (122.9 ± 4%); J-CD-RU (132.8 ± 5.3%)]. The J-HFD-VEH group displayed impaired LTP [J-HFD-VEH (97.5 ± 2.2%)] in accordance with experiment 2 (Figure 3). The J-HFD-RU has intact LTP [J-HFD-RU (123.5 ± 2.31%)] comparable to the CD fed groups.

However, it can be said that the juveniles have a different response to systemic GR antagonist. Specifically, inhibition of GRs impairs the prefrontal LTP in adult controls without affecting juvenile controls. The J-HFD-RU has intact LTP hinting that such inhibition may rescue the inhibiting effect of HFD on LTP in the mPFC.

### Experiment 4: Intra mPFC Microinjection of GR Antagonist Reverses HFD-Induced LTP Impairment in Juveniles but Had no Effect on Adults

Based on the findings from the above experiment that systemic RU-486 treatment differential effect on LTP in juveniles and adults, we aimed in this experiment to address the specific role of prefrontal GRs in mediating the effect of HFD on mPFC-LTP. We tested the following groups: VEH (J-CD-VEH, *n* = 5; J-HFD-VEH, *n* = 6; A-HFD-VEH, *n* = 4; A-CD-VEH, *n* = 5) or RU-486 injections (J-CD-RU, *n* = 7; J-HFD-RU, *n* = 6; A-CD-RU, *n* = 6; A-HFD-RU, *n* = 6).

The baseline amplitude and the stimulation intensity required to produce comparable baseline signals were not affected by Age, Diet, or Drug [*F*(1, 37) < 1].

Analysis of variance with repeated measures on the time points following TBS showed significant effects of Diet [*F*(1, 37) = 38.116, *p* < 0.001], Age [*F*(1, 37) = 16.041, *p* < 0.001] and Diet X Drug interaction [*F*(1, 37) = 40.300, *p* < 0.001] but without an effect of Drug *F*(1, 37) = 0.039, n.s.].

**FIGURE 6:**
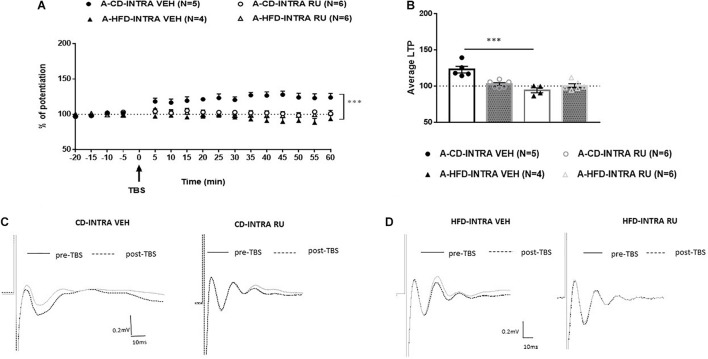
Effects of local intra-mPFC GR blockade on prefrontal LTP in HFD- and CD-fed adults. **(A)** Normalized fEPSPs recorded *in vivo* before and after TBS of the BLA-mPFC pathway. Each data point shows the average fEPSPs of the preceding 5 min. **(B)** Average LTP shown in panel A over the 60 min after TBS. **(C,D)** Representative average waveforms before and after TBS Horizontal bar = 10 ms; vertical bar = 0.2 mV. For adult animals locally microinjected with GR antagonist RU-486 (RU) or vehicle 30 min prior to TBS. RU treatment had no effect on HFD-impaired LTP in HFD-fed animals, but impaired LTP in control CD-fed animals. Two-way ANOVA with repeated measures on the 12 time points after TBS showed significant effects of Diet (^∗∗∗^*p* < 0.001) and Diet × Drug interaction (^∗∗∗^*p* < 0.001), without an individual effect of Drug. *Post hoc* analysis showed a significant difference in CD-fed animals between vehicle and RU-486 groups (^∗∗∗^*p* < 0.001), and no significant difference between HFD-treated groups. Error bars show standard error of the mean (SEM).

To understand the Diet × Drug interaction, each age was analyzed separately. In adults ANOVA with repeated measures indicated significant effects of Diet [*F*(1, 17) = 23.019; *p* < 0.001] and interaction between Diet and Drug [*F*(1, 17) = 17.644; *p* < 0.001] without an effect of Drug [*F*(1, 17) = 4.705, n.s; Figure 6]. *Post hoc* analysis showed in CD fed animals, a significant difference between RU-486- and VEH group [*F*(1, 9) = 18.688, *P* = 0.002]. Whereas, the CD-VEH group showed intact potentiation, RU-486 treated group did not show potentiation [A-CD-VEH (123.2 ± 4.4%); A-CD-RU (103.5 ± 2.2%)]. In HFD animals, no differences were observed between vehicle and RU-486 [*F*(1, 8) = 2.364, n.s] and did not show any potentiation [A-HFD-VEH (94.1 ± 4.5%); A-HFD-RU (100.3 ± 3.3%)].

**FIGURE 7:**
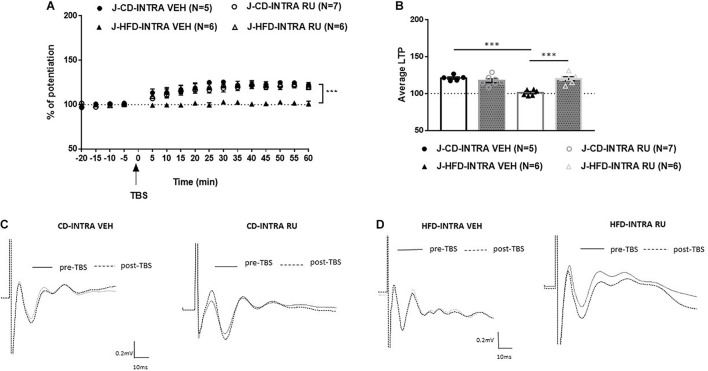
Effects of local intra-mPFC GR blockade on prefrontal LTP in HFD- and CD-fed juveniles. **(A)** Normalized fEPSPs recorded *in vivo* before and after TBS of the BLA-mPFC pathway. Each data point shows the average fEPSPs of the preceding 5 min. **(B)** Average LTP shown in panel A over the 60 min after TBS. **(C,D)** Representative average waveforms before and after TBS Horizontal bar = 10 ms; vertical bar = 0.2 mV. For juvenile animals locally, microinjected with RU-486 (RU) or vehicle 30 min prior to TBS. RU treatment rescued HFD-impaired LTP in HFD-fed animals, and had no effect on control CD-fed animals. Two-way ANOVA with repeated measures on the 12 post-TBS time points showed significant effects of Diet (^∗∗∗^*p* < 0.001), Drug (^∗∗∗^*p* < 0.001), and Diet × Drug interaction (^∗∗∗^*p* < 0.001). *Post hoc* comparison showed a significant difference between vehicle and RU-486-treated HFD-fed groups (^∗∗∗^*p* < 0.001), but no significant difference between the CD-fed groups. Error bars show standard error of the mean (SEM).

In the juvenile animals, ANOVA with repeated measures showed significant effects of Diet [*F*(1, 20) = 13.780, *p* < 0.001], Drug [*F*(1, 20) = 10.783, *p* < 0.001] and of interaction between Diet and Drug [*F*(1, 20) = 23.190, *p* < 0.001; Figure 7]. *Post hoc* analysis showed that in CD fed animals, no differences were observed between vehicle and RU-486 [*F*(1, 10) = 1.19, n.s] and both groups expressed potentiation [J-CD-VEH (120.8 ± 3.9%); J-CD-RU (117.2 ± 2.2%)]. Whereas, in HFD fed animals, a significant difference was observed between RU-486 and vehicle group [*F*(1, 10) = 32.280, *P* < 0.001], while HFD-VEH did not show any potentiation, RU-486 group showed intact potentiation [J-HFD-VEH (100.4 ± 1.5%); J-HFD-RU (119.5 ± 2.6%)].

Thus, these results indicate that GR antagonist locally in the IL-mPFC rescues HFD impairment of mPFC-LTP in juveniles only. Local inhibition of GRs with RU-486 impairs prefrontal LTP in CD-fed adults only.

### Experiment 5: Acute HFD Induces Changes in PFC GR mRNA Expression Level in Juveniles but Not Adults

**FIGURE 8:**
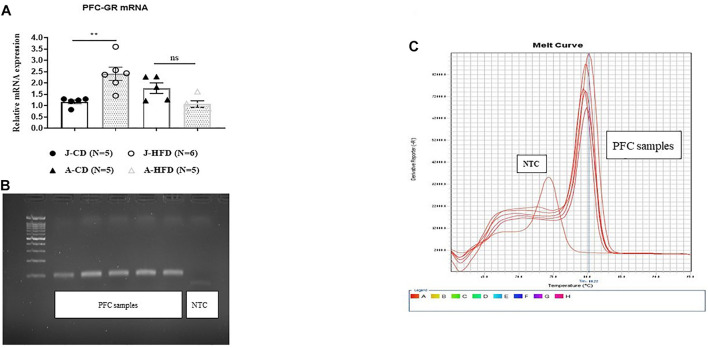
Effects of acute HFD exposure on prefrontal cortex GR mRNA expression. **(A)** Prefrontal cortex (PFC) GR mRNA of juvenile (PND 28) and adult (PND 67) animals immediately after completing a 7-day HFD or CD regimen was quantified using RT-qPCR. Error bars show SEM. Univariate ANOVA analysis by Age and Diet of mRNA expression levels showed a significant effect of Age × Diet interaction (^∗∗∗^*p* < 0.001), but no significant individual effects of Age (n.s) or Diet (n.s). *Post hoc* analysis showed significant differences in PFC GR mRNA expression between CD- and HFD-fed juveniles (^∗∗^*p* < 0.01), but not adults (n.s.). **(B**,**C)** Representation of gel image and dissociation curve. Error bars show standard error of the mean (SEM).

As the GR antagonist modulated prefrontal LTP differently in HFD and CD-fed juveniles and adults, we next examined whether these groups differed in GR mRNA expression by using RT-qPCR (Figure 8). The groups were as following: [J-CD (*n* = 5), J-HFD (*n* = 6), A-CD (*n* = 5), A-HFD (*n* = 5)]. Univariate ANOVA analysis by Age and Diet of mRNA expression levels showed a significant effect of Age × Diet interaction [*F*(1, 20) = 13.165, *p* < 0.001], but no significant individual effects of Age [*F*(1, 20) = 1.750, n.s] or Diet [*F*(1, 20) = 1.12, n.s]. *Post hoc* analysis showed a significant effect of Diet on PFC GR mRNA expression in juveniles [*F*(1, 11) = 8.146, *p* < 0.01], but not adults [*F*(1, 10) < 1, n.s.]. In juveniles, HFD showed an increase in the GR mRNA level as compared to CD (CD: 1.173 ± 0.088; HFD: 2.409 ± 0.29). Whereas, in adults, no differences were observed between the two groups (CD: 1.77 ± 0.23; HFD: 1.07 ± 0.14). These results indicate that acute HFD exposure is associated with an increase in GR mRNA expression in juveniles only.

### Experiment 6: Intra-mPFC Microinjection of GR Agonist RU-28362 Reverses HFD-Induced LTP Impairment in Adults Without Affecting Controls

**FIGURE 9:**
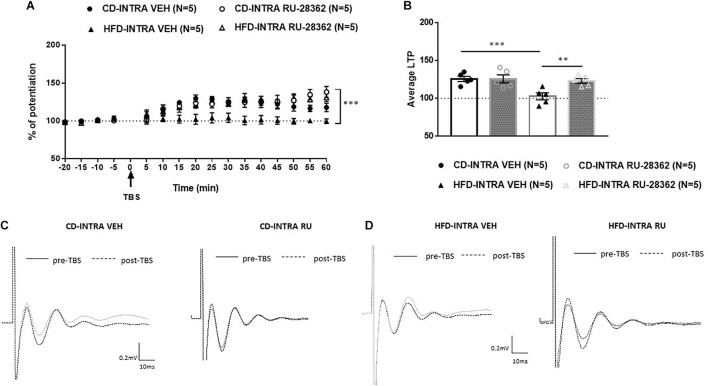
Effects of intra-mPFC GR activation on prefrontal LTP in HFD- and CD-fed adults. **(A)** Normalized fEPSPs recorded *in vivo* before and after TBS of the BLA-mPFC pathway. Each data point shows the average fEPSPs of the preceding 5 min. **(B)** Average LTP shown in panel A over the 60 min after TBS. **(C,D)** Representative average waveforms before and after TBS Horizontal bar = 10 ms; vertical bar = 0.2 mv. For adult animals locally, microinjected with GR agonist RU-28362 or vehicle 30 min prior to TBS. Agonist treatment rescued HFD-impaired LTP in HFD-fed animals, and had no effect on CD-fed animals. Two-way ANOVA with repeated measures on the 12 post-TBS time points showed significant effects of Diet (^∗^*p* < 0.05), Drug (^∗∗∗^*p* < 0.005), and Diet × Drug interaction (^∗∗^*p* < 0.01). *Post hoc* comparisons showed significant differences between RU-28362 and vehicle-treated HFD-fed animals (^∗∗^*p* < 0.01), and no significant differences between CD-fed animals.

We showed that systemic or local blockade of GRs impairs prefrontal LTP in CD-fed adults, but has no impact in HFD-fed adults and we did not find any difference in the mRNA level in HFD fed adults. These may suggest that GR activity in the mPFC modulates the induction of LTP in both CD and HFD adults. Therefore, we sought to examine whether GR activation would affect the prefrontal LTP of HFD-fed adult animals. To locally activate GRs, we microinjected the GR agonist RU-28362 or vehicle into the IL-mPFC of CD or HFD-fed adult animals 30 min before TBS and assessed LTP (Figure 9). The groups were as following: RU-28362 (A-CD-RU, *n* = 5; A-HFD-RU, *n* = 5) or VEH (A-CD-VEH, *n* = 5; A-HFD-VEH, *n* = 5). The baseline amplitude and the stimulation intensity necessary to elicit the baseline response did not show significant effects of Diet or Drug [*F*(1, 16) < 1 for both]. ANOVA with repeated measures on the 12 post-TBS time points [Diet (CD, HFD); Drug (VEH, RU-28362)] revealed significant effects of Diet [*F*(1, 16) = 5.612, *p* < 0.05], Drug [*F*(1, 16) = 8.750, *p* < 0.005], and Diet × Drug interaction [*F*(1, 16) = 5.667, *p* < 0.05], suggesting that the GR agonist has different effects on the HFD- and CD-fed groups.

To understand the Diet × Drug interaction, each Diet was analyzed separately. *Post hoc* analysis showed a significant difference between the HFD-RU-28362- and HFD-VEH animals [*F*(1, 8) = 14.146, *p* < 0.05]: HFD-VEH group did not show any potentiation (102.6 ± 4.22%), whereas this was rescued in the RU-28362-treated group, which showed intact potentiation (123.07 ± 3.12%). In CD-fed adults, RU-28362 did not have an effect [*F*(1, 8) = 0.168, n.s], as both vehicle-treated (CD-VEH: 125.7 ± 3.35%) and RU-28362-treated groups expressed intact potentiation (CD-RU-28362: 125.8 ± 5.22%). These data suggest that GR agonist treatment reverses the HFD-induced LTP impairment without affecting controls.

## Discussion

Our present study demonstrates striking differences between the role of GRs in HFD-induced modulation of prefrontal LTP in adults and juveniles. Following acute exposure to HFD, we observed an impairment in LTP in both juveniles and adults. Further, blocking GRs both systemically and locally in the mPFC rescued HFD-induced LTP impairment in juveniles, but not adults. In contrast, this treatment impaired LTP in CD-fed adults but not CD-fed juveniles. Impairments in the prefrontal LTP following HFD are associated with an increase in the levels of GR expression in the mPFC in juveniles only. Further, GR agonists into mPFC in adults rescued HFD-induced impairment in LTP. Together, these results clearly show that even though HFD induces LTP impairment at both ages, the mechanisms that may mediate these impairments are likely dissimilar.

### Age and Diet Effects on Prefrontal LTP

Although the majority of studies to date have addressed the effects of chronic exposure to HFD in the hippocampus of adult animals (Valladolid-Acebes et al., 2013; Boitard et al., 2014; Hsu et al., 2015; Vinuesa et al., 2016), the effects of HFD on the mPFC and the amygdala remain less well understood. Recent work has shown that HFD can indeed affect various forms of cognitive functions that rely on the mPFC (Kanoski et al., 2007; Bocarsly et al., 2015; Yaseen et al., 2018). These studies reported adverse effects of HFD on developing mPFC and amygdala during adolescence, thus highlighting the sensitivity of these regions to the consumption of obesogenic diets (Francis and Stevenson, 2013; Guillemot-Legris and Muccioli, 2017). In particular, the majority of these investigations revealed that chronic HFD affects a variety of synaptic functions within the mPFC, including glutamatergic neurotransmission and plasticity. However, acute HFD-related alteration in the mPFC at the cellular level in juveniles and adults remain poorly described so far. Thus, we hypothesized acute HFD would affect mPFC-LTP in an age-dependent manner due to the ongoing maturation of the mPFC. First, we investigated the effects of HFD consumption for 7 days and established that acute consumption of HFD that is independent of body weight change leads to impaired mPFC LTP in adults as well as juveniles. These results in juveniles are consistent with our previous study in juveniles (Yaseen et al., 2018), suggesting that a cautious dietary equilibrium during this sensitive period is crucial for reaching the full volume of adult prefrontal functions (Reichelt et al., 2015a).

This impairment in adults following the consumption of HFD, is consistent with recent studies that reported a reduced dendritic spine density in mPFC (Dingess et al., 2017), dendritic length in the basal arbors of the BLA (Janthakhin et al., 2017), and GABA concentration in the PFC of rats following chronic HFD consumption (Sandoval-Salazar et al., 2016). These data on the impairments in mPFC plasticity in the young adult animal following consumption of HFD are not in line with the effects observed in the hippocampus following similar exposure. We have previously shown that similar exposure resulted in enhanced hippocampal functions and plasticity in the CA1 (Khazen et al., 2019). It is also established that acute exposure to HFD impairs hippocampus- and amygdala-dependent functions in 24 month-old rats but not in young adults [same age used in this study (Spencer et al., 2017)]. These together suggest that the way HFD affects brain functions is brain region-dependent. Taken together, our results support that even a short period of HFD is detrimental to health, particularly to PFC-dependent functions. It is important to note that in the present study we did not use mPFC-dependent memory task to address the effects of HFD, future studies should address this. It is worthy to mention that in the present study we used different protocols for the induction of LTP in adults and juveniles. We recently reported that juvenile animals differ in the ability to induce LTP in the medial prefrontal cortex compared to adults (Schayek and Maroun, 2015). Differences in the stimulation frequency required to obtain LTP in juveniles and adults were previously reported by us and others (Zitman and Richter-Levin, 2013; Khazen et al., 2018; Yaseen et al., 2018). The protocol of 100 HZ was not sufficient to induce LTP in the juvenile animals, thus we used the 200 HZ for this group. However, when applied to adult animals the 200 HZ protocol resulted in potentiation levels that were significantly different from the juveniles. In addition, we could not observe an effect of HFD as both the HFD and CD group showed potentiation. Thus the 200 HZ protocol could not be applied to the adult group as it led to a ceiling effect.

### The Effect of Glucocorticoids on HFD-Induced mPFC-LTP Changes

Obesogenic diets have been shown to contribute to HPA axis dysregulation in rats (Avignon et al., 2012; Abildgaard et al., 2014; Boitard et al., 2015; McNeilly et al., 2015; Kalyan-Masih et al., 2016; Sobesky et al., 2016; Vega-Torres et al., 2018; Khazen et al., 2019), and GRs, which are located in both the hippocampus and the prefrontal cortex, may contribute to the modulation of the HPA axis (Finsterwald and Alberini, 2014). Dietary macronutrient content is an important determinant of metabolic health independent of changes in body weight (Hu et al., 1997) and this may be partly mediated by changes in glucocorticoid action. Further, it is known that glucocorticoids respond acutely to changes in nutritional status (Benedict et al., 2005). Therefore, this study investigated the effect of GRs on the modulation of prefrontal plasticity in juveniles and adults following acute exposure to HFD. Systemic and intra-mPFC injections of the GR antagonist RU-486 had age-dependent effect. Specifically, RU-486 rescued HFD-impaired LTP in juveniles (Figures 5, 7), but had no effect on HFD-fed adults (Figures 4, 6). Interestingly, though RU-486 had no effect on control CD-fed juveniles (Figures 5, 7), it impaired LTP in CD-fed adults (Figures 4, 6). However, the precise mechanism for this inhibition in adult controls is yet to be studied.

Further, we found that local GR activation with RU-28362 was able to rescue HFD-impaired LTP in adults, contrary to the systemic or local injection of GR antagonist, which failed to induce LTP in the mPFC (Figures 4, 6). Also unlike the GR antagonist, the GR agonist RU-28362 did not have any effect on CD-fed adults (Figure 9). Therefore, we posited that this age-dependent modulation of GRs following brief exposure to HFD might occur as a result of alterations in glucocorticoid negative feedback to the HPA axis (Herman et al., 2012; Gjerstad et al., 2018; Kurek et al., 2018). Notably, blockade of GRs by the GR antagonist RU-486 also restored impaired LTP in mPFC of stress-induced rats (Yang et al., 2004; Kavushansky et al., 2006; Krugers et al., 2006; Mailliet et al., 2008). The effect of blockade of LTP in CD in adult animals may suggest that there is an optimal level of Cort that is needed for intact LTP. Application of GR agonist rescued the LTP in HFD fed animals but normalized LTP in adult controls. These findings correlate with previous published data that have shown the contrasting effect of GR agonist and antagonist on LTP and synaptic plasticity (Diamond et al., 1992; Pavlides et al., 1996; Avital et al., 2006; Fontanez-Nuin et al., 2011). Taken together, the evidence seems to support our previously stated hypothesis, an optimal level of glucocorticoid receptor activity is required for proper induction of LTP and deviation from this optimum in both dictions has deleterious effects on plasticity. We further determined the age-dependent mRNA expression levels in these rats. We found GR mRNA levels in the PFC to be elevated only in HFD juveniles but not adults (Figure 8), suggesting that HFD may attenuate HPA axis function which is modulated by a feedback regulation of glucocorticoid and adrenocorticotropic hormone (ACTH) through the hippocampus, paraventricular nucleus, and pituitary gland (Yam et al., 2015). Alterations in GR expression have also been associated with impaired regulation of the HPA axis that may contribute to metabolic and physiological obesity-related changes (Tannenbaum et al., 1997). Therefore, it can be speculated that the neuroplastic alterations in juvenile HFD-fed rats may be attributed to sensitivity of the HPA axis induced by HFD. We did not measure the plasma corticosterone level in this study because we recently reported that acute exposure to HFD led to an increase in the plasma corticosterone in juveniles only but had no effect on adults (Khazen et al., 2019). These evidence suggest that greater HPA axis dysregulation occurs in the juveniles. Thus, when we reported that the effects of HFD on LTP were the same in the two groups of age, we assumed that GRs blockade will rescue, yet it seems that there is a need for optimal level of corticosterone for intact levels of LTP. This may explain why in juveniles blockade rescued LTP while in adults the agonist reversed the levels of LTP which are correlated with previous literature (Wiegert et al., 2006; Fontanez-Nuin et al., 2011). Taken together, we posit that GR modulation in combination with acute HFD are critical for the induction of age-dependent deleterious effects of GRs on prefrontal LTP. However, more studies are required to elucidate possible mechanisms by which the interplay between GRs and acute HFD affects neurophysiological mechanisms in the PFC.

## Conclusion

In conclusion, to the best of our knowledge, this is the first study comparing adult and juvenile animals that addresses the effects of acute HFD and GR modulation on the magnitude of LTP in the mPFC. The results of the present study show that LTP impairment in the mPFC occurs in a GR-dependent manner in adults and juveniles. These results suggest that during juvenility, HFD activates stress related system that is not present in adulthood. These findings may prove valuable for introducing novel age-specific strategies to treat stress-related disorders such as anxiety and depression reported in obese patients.

## Data Availability Statement

The original contributions presented in the study are included in the article/supplementary material, further inquiries can be directed to the corresponding author/s.

## Ethics Statement

The animal study was reviewed and approved by University of Haifa Ethical Committee for animal experimentation.

## Author Contributions

KS and MM designed the experiments. KS and TR performed the experiments and analyzed the data. KS, NM, and MM wrote the manuscript. All authors contributed to the article and approved the submitted version.

## Conflict of Interest

The authors declare that the research was conducted in the absence of any commercial or financial relationships that could be construed as a potential conflict of interest.

## Publisher’s Note

All claims expressed in this article are solely those of the authors and do not necessarily represent those of their affiliated organizations, or those of the publisher, the editors and the reviewers. Any product that may be evaluated in this article, or claim that may be made by its manufacturer, is not guaranteed or endorsed by the publisher.
